# Hypersensitivity pneumonitis with *Paecilomyces* as suspected inciting antigen

**DOI:** 10.1016/j.rmcr.2024.102013

**Published:** 2024-03-21

**Authors:** Masayuki Watanabe, Ryo Okuda, Yuriko Ishida, Shinobu Sagawa, Tatsuya Muraoka, Satoshi Ikeda, Tomoe Sawazumi, Tamiko Takemura, Eri Hagiwara, Takashi Ogura

**Affiliations:** a Department of Respiratory Medicine, Kanagawa Cardiovascular and Respiratory Center, Yokohama, Japan; b Department of Pathology, Kanagawa Cardiovascular and Respiratory Center, Yokohama, Japan

**Keywords:** Acute hypersensitivity pneumonitis/ antigen avoidance test, Fungi/ mold/ *Paecilomyces*/ persistent cough

## Abstract

A 73-year-old man visited our hospital for persistent cough. Chest high-resolution CT (HRCT) showed infiltration shadows in lower lobes and diffuse ground glass opacities in the upper lobes. Blood tests showed elevated white blood cell, C-reactive protein, surfactant protein D, and Krebs von den Lungen-6 levels. After an antigen avoidance test, his HRCT and blood test findings improved; we diagnosed him with hypersensitivity pneumonitis (HP). A culture of the rotting interior walls within his home revealed *Paecilomyces*, which we believe caused his HP. Given the few patients with *Paecilomyces-*induced HP, systematic approach was important to identify the inciting antigen.

## 1 Introduction

Hypersensitivity pneumonitis (HP) is a complex syndrome caused by an immune response to the inhalation of a wide variety of organic particles. Although the most common inciting antigens of HP are avian proteins and fungi, several cases related to occupational and living conditions have also been reported. Fungi such as *Alternaria*, *Aspergillus*, *Cryptostroma*, *Penicillium*, *Pullularia*, *Rhodotorula*, and *Trichosporon* are among the most common causes of HP [1,2]. *Paecilomyces* is a putrefactive fungus which inhabits decaying plants, soil, wood, and food. Several reports have suggested that *Paecilomyces* can cause fungal pneumonia [3]; however, only a few reports have been available on HP caused by *Paecilomyces* [[4], [5], [6], [7], [8]]. In the current report, we detail our diagnosis of HP due to *Paecilomyces* through a systematic approach.

## 2 Case presentation

**Fig. 1:**
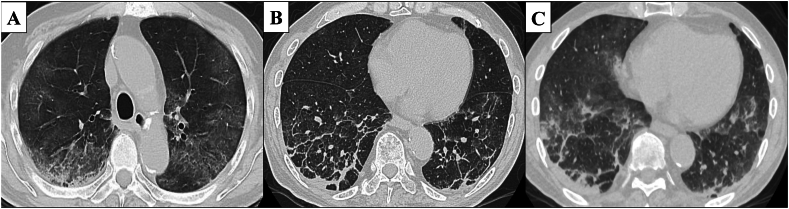
HRCT on admission Chest HRCT image on admission. (A) Diffuse ground glass opacities predominantly in the upper lobes. (B) Infiltration shadows were seen in the bilateral lower lobes. (C) Air trapping was observed in expiratory CT.

**Fig. 2:**
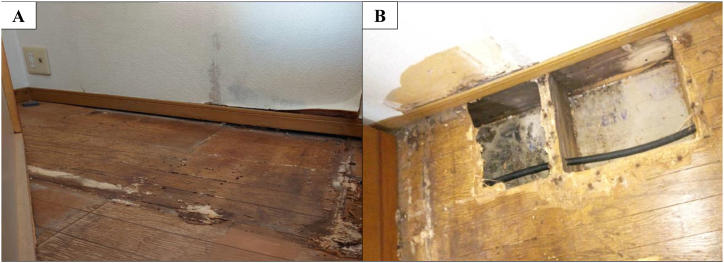
Image of patient's house Wood rot was observedon (A) floor and (B) wall.

**Fig. 3:**
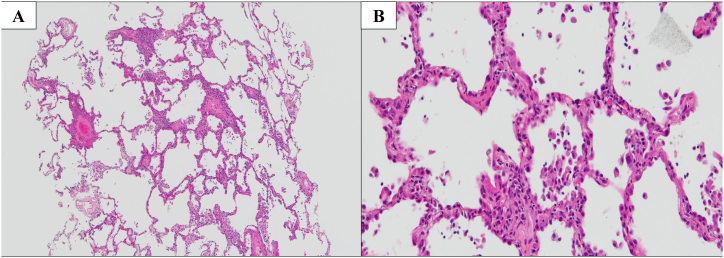
Pathologic findings Hematoxylin-Eosin Stain in right lower lobe B⁹b, showing lymphocyte and plasma cell infiltration in the airway center. (A) Weak magnification: 40×, (B) Strong magnification: 200×.

**Fig. 4:**
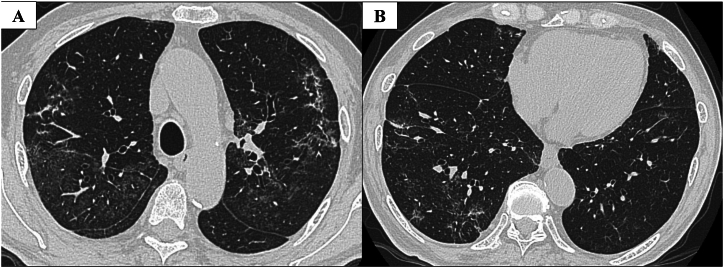
HRCT after 31 days from antigen avoidance test (A) The ground glass opacities in the bilateral upper lobes were improved. (B) The ground glass opacities in bilateral lower lobes were also improved.

Our case involved a 73-year-old man who was a former company employee. He had hypertension as a comorbidity but was otherwise healthy without any other notable medical history. He drinks alcohol around three times a week and had no history of smoking, drug or food allergies, or bird breeding. He had been suffering from a persistent cough since June 2022, which prompted him to visit his local doctor in early September. Given that no abnormalities were noted on chest X-ray, only antitussive medication was prescribed. However, his cough worsened and his oxygen saturation (SpO₂) fell below 90% at rest using an arterial blood oxygen saturation monitor at home. Hence, he was sent to our hospital on September 14, 2022 for a thorough examination of pneumonia. A chest high-resolution CT (HRCT) at admission showed bilateral lower lobe infiltration shadows, diffuse ground glass opacities predominantly in the upper lobes, and air trapping during exhalation CT (Fig. 1). The several parameters of blood tests were elevated: white blood cells 10010 /μL (reference range: 3300–8600 /μL), C-reactive protein (CRP) 8.96 mg/dL (reference range: 0–0.30 mg/dL), and surfactant protein D (SP-D), 244.8 ng/mL (reference range: 0–109.9 ng/mL). Specific Immunoglobulin G testing for avian antibody was negative. *Aspergillus* antibody and cytomegalovirus antigen tests came back were negative. We suspected HP based on the living environment (Fig. 2), including the rotting walls and household items in his room, ground glass opacities predominantly in the upper lobes on chest HRCT, and elevated SP-D value. Transbronchial lung cryobiopsy from the right B⁹ a,b and bronchoalveolar lavage (BAL) from the right B⁴ were performed. Histopathology revealed a central airway infiltrate of lymphocytes and plasma cells, with small intraluminal organization in the alveolar space and some macrophage clusters. No obvious granuloma formation was observed (Fig. 3). The leukocyte fraction in the BAL fluid was as follows: Mφ 17.0%, lymphocytes 80%, neutrophils 2.5%, and eosinophils 0.5%. According to the 2020 American Thoracic Society/ Japanese Respiratory Society/ The Latin American Thoracic Association HP guidelines, our patient was determined to be a definite non-fibrotic HP case. An antigen avoidance test was subsequently performed at our hospital. Although the patient required 4 L/min of oxygen at admission, oxygen administration was terminated on the 9th day after avoidance test. Moreover, his white blood count and CRP decreased from 10010 /μL to 9720 /μL and 8.96 to 2.64 mg/dL on 9 days after avoidance test, respectively (Table 1). Chest HRCT findings performed 9 days after the antigen avoidance test showed a decrease in some of the ground glass opacities. Respiratory function tests performed on the 9th and 31st days after the antigen avoidance test showed improvement in his forced vital capacity (FVC) from 102% to 130%. 31st after the antigen avoidance test decreased in his serum CRP (0.10 mg/dL). On HRCT finding, ground glass opacities were mostly improved; however, some reticular shadows remained, suggesting a diagnosis of fibrotic HP caused by *Paecilomyces* (Fig. 4). The inciting antigen was considered to have existed in his home environment. Given that the home environment of the patient was suspected to be antigenic, his family scraped off some of the rotting walls and furniture in his home and submitted them for culture, which led to the detection of *Paecilomyces*. The results of antibody precipitation reaction were negative for *Trichosporon*, *Cephalosporium*, *Aspergillus*, *Penicillium*, and *Cladosporium*. He was ultimately diagnosed with HP caused by *Paecilomyces* and was discharged from the hospital after the rotting interior walls and furniture in his home, which could have been the source of *Paecilomyces*, were removed and replaced. Respiratory function tests performed 2 months after discharge showed an improvement in his FVC to 135%, while chest HRCT performed 4 months after discharge showed a decrease in diffuse ground glass opacities and serum CRP (0.20 mg/dL).

**Table 1:** Changes of laboratory data and pulmonary function test.

	On admission	9 days after avoidance test	31 days after avoidance test	2 months after discharge	4 months after discharge
WBC, μL	10010	9720	6720	8840	9160
LDH, U/L	194	200	185	173	167
CRP, mg/dL	8.96	2.64	0.10	0.33	0.20
KL-6, U/mL	376	650	827	491	320
FVC, %	NA	102	130	135	NA
DLco, %	NA	75	91	NA	NA
SpO₂, % (Oxgen requirement, L/min)	96 (3)	94 (0)	97 (0)	98 (0)	97 (0)

WBC, white blood cell; LDH, lactate dehydrogenase; CRP, C-reactive protein; KL-6, Krebs von den Lungen-6; FVC, forced vital capacity; DLco, diffusing capacity of the lung for carbon monoxide; SpO_2_, oxygen saturation; NA, not available.

## 3 Discussion

We herein detail our experience with a patient who developed HP probably due to the inhalation of *Paecilomyces* that grew on the rotting interior walls and furniture in his home. *Paecilomyces* is a fungus commonly found in the environment that is resistant to high temperatures and low oxygen and is known to cause spoilage of canned and bottled fruits and fruit juices [2]. *Paecilomyces* can also cause opportunistic infections in immunocompromised animals. The genus *Paecilomyces* includes several species, with *Paecilomyces variotii* and *Paecilomyces lilacinus* being considered the most common causes of infection. *P. variotii* has been reported to cause pneumonia, intraocular inflammation, sinusitis, peritonitis, and soft tissue infections, whereas *P. lilacinus* causes intraocular lens implant infections and invasive soft tissue infections. Treatment for conditions caused by the aforementioned fungi includes voriconazole and posaconazole [3]. The HP in the current patient was caused by inhalation, not by direct pneumonia caused by *Paecilomyces*. There have been only five reported cases of HP due to *Paecilomyces* exposure, highlighting the rarity of this condition (Table 2). In four out of the five patients, wood rot or wood products were the source of the antigen, and patients varied in terms of country, sex, and age. In general, when HP is diagnosed in patients who have had contact with wood, the inciting antigens often include *Aspergillus*, *Penicillium*, *Cryptostroma corticale*, *Alternaria*, and *Trichoderma* [5]. Aside from wood involvement, *Aspergillus fumigatus* has been reported to cause farmer's lung, whereas genera *Thermoacinomyces* and *Trichoderma* have been found to cause humidifier lung, among others. In Japan, HP caused by a fungus is also known as summer-type hypersensitivity pneumonitis (SHP), which is caused by *Trichosporon* present in moldy wood within Japanese houses. Epidemiological studies of HP in Japan had reported that SHP accounted for 74% of the cases from 1980 to 1989 and 70% of the cases from 1990 to 1999 [9]. Although the onset of the disease in our patient occurred in the summer, he tested negative for the anti-*Trichosporon asahii* antibody and precipitating antibody against *Trichosporon*. After a detailed medical history and living environment interview, antigen avoidance test, and specific ImmunoglobulinG testing, we determined that the inciting antigen in this patient was *Paecilomyces*. After the inciting antigen was removed from his environment, his condition remained improved in current case. Given that the identification of the inciting antigen leads to improved prognosis, emphasis should be placed on uncovering the antigen inciting HP through environmental surveys around the home, antigen avoidance test, and precipitating antibody test.

**Table 2:** Summary of hypersensitivity pneumonitis from exposure to *Paecilomyces*.

Authors	Number of cases	Sex	Age	Exposure	Countries	Years	nfHP or fHP
Kauppinen et al. [4]	35	Unknown	Unknown	Fermented pulp	Finland	1983	Unknown
Dykewicz et al. [5]	1	Male	28	Wood chips	America	1988	Unknown
Bryant et al. [6]	12	Unknown	Unknown	Rotten wood	Australia	1991	Unknown
Veillette et al. [7]	13	Unknown	Unknown	Dry wood processing plant	Canada	2006	nfHP
Hara et al. [8]	1	Male	57	Oil fan heater	Japan	2006	nfHP

nfHP, nonfibrotic hypersensitivity pneumonitis; fHP, fibrotic hypersensitivity pneumonitis.

## 4 Conclusion

*Paecilomyces* rarely causes HP. However, given that *Paecilomyces* itself is commonly found in decaying wood, inhalation exposure can easily cause HP. In our patient, we were able to identify the antigen inciting HP within the home environment. A combination of medical interviews, home investigations, fungal cultures, and antigen testing can help with the identification of the inciting antigen in patients with HP.

## Funding

There was no funding for current study.

## CRediT authorship contribution statement

**Masayuki Watanabe:** Writing – original draft. **Ryo Okuda:** Writing – review & editing. **Yuriko Ishida:** Writing – review & editing. **Shinobu Sagawa:** Writing – review & editing. **Tatsuya Muraoka:** Writing – review & editing. **Satoshi Ikeda:** Writing – review & editing. **Tomoe Sawazumi:** Writing – review & editing. **Tamiko Takemura:** Writing – review & editing. **Eri Hagiwara:** Writing – review & editing. **Takashi Ogura:** Writing – review & editing.

## Declaration of competing interest

None.
